# Active Polypropylene-Based Films Incorporating Combined Antioxidants and Antimicrobials: Preparation and Characterization

**DOI:** 10.3390/foods10040722

**Published:** 2021-03-29

**Authors:** Seyed Hadi Peighambardoust, Seyedeh Homa Fasihnia, Seyed Jamaleddin Peighambardoust, Mirian Pateiro, Rubén Domínguez, José M. Lorenzo

**Affiliations:** 1 Department of Food Science, College of Agriculture, University of Tabriz, Tabriz 5166616471, Iran; h_fasihnia@tabrizu.ac.ir; 2 Faculty of Chemical and Petroleum Engineering, University of Tabriz, Tabriz 5166616471, Iran; j.peighambardoust@tabrizu.ac.ir; 3 Centro Tecnológico de la Carne de Galicia, Rúa Galicia N° 4, Parque Tecnológico de Galicia, San Cibrao das Viñas, 32900 Ourense, Spain; mirianpateiro@ceteca.net (M.P.); rubendominguez@ceteca.net (R.D.); 4 Área de Tecnología de los Alimentos, Facultad de Ciencias de Ourense, Universidad de Vigo, 32004 Ourense, Spain

**Keywords:** active packaging, polypropylene, BHA, BHT, sorbic acid, mechanical properties

## Abstract

Development of polypropylene (PP) films incorporating antioxidant-antimicrobial agents can inhibit microbial growth and reduce undesirable deteriorating reactions and can preserve the quality of food. This study was aimed to use a combination of sorbic acid (SA), butylated hydroxyanisole (BHA), and butylated hydroxytoluene (BHT) to provide a synergistic effect at their reduced concentrations. A Combination of the additives was more effective in enhancing mechanical properties compared to their single state in film composition. The PP-2%SA-3%BHA film (T_3_) had the highest tensile strength (17.9 MPa) and the lowest elongation at break (7.1%) than other films. The fourier-transform infrared (FTIR) proposed physical mixing of active additives within PP-matrix. Scanning electron microscopy showed uniform dispersion of the additives in PP-2%SA-1%BHT-1%BHA film (T_4_) compared to others. BHT containing films decreased the storage and loss moduli leading to weakening of film viscoelastic behaviour and reducing film melting point. The prepared active films showed higher antioxidant activity than control PP-film following an order of T_4_ > T_2_ > T_3_ corresponding to DPPH radical scavenging values of 89.1, 83.4 and 79.1%, respectively. All active films inhibited gram-negative and gram-positive bacteria growth. The results of this study indicated that the prepared active films possess desirable mechanical, thermal, antioxidant and antimicrobial properties enabling their use in food packaging.

## 1. Introduction

Microbial contamination and oxidation are two major factors deteriorating food quality after processing [1,2]. As a result, antimicrobial and antioxidant substances are being used to maintain food quality and extend the shelf-life of food products [3,4]. However, incautious use of chemical preservatives may show toxic effects in food products [5,6]. It has been reported that certain chemical preservatives show signs of genotoxicity when used at higher concentrations and may induce various type of chromosomal abnormalities [7]. In the food industry, synthetic antioxidants and antimicrobial substances are widely used to preserve products against oxidation reactions and microbial contaminations [8,9]. In an early report by Leslie et al. [10] it was shown that butylated hydroxyanisole (BHA) and butylated hydroxytoluene (BHT) can become cytotoxic leading to myocardial cultured cell injury and lysis at higher concentrations after long exposure time. In recent investigations, using flow cytometry analysis, the toxic effect of BHT on rat thymocytes cells was also proven [11]. Likewise, animal studies exhibited BHA-mediated liver toxicity and retardation in reproductive organ development [12]. As a result, the possible toxicity and carcinogenicity of synthetic antioxidants may counteract their direct consumption in food formulations [13].

Active packaging can minimize the direct use of chemical preservatives in food formulation, and thus, is gaining increased attention in extending food shelf-life [14,15,16,17]. A part from safety concerns mentioned above, from technological point of view, the direct application of antioxidant and antimicrobial agents to the bulk of food may hinder their activity because of possible interactions with other ingredients in food [18,19,20,21,22]. Hereof, designing active packaging films with controlled release of active substances could protect food from spoilage [23]. Besides, active films can favour reduction of additives concentrations, while offering the same level of protection, which can be achieved by direct incorporation of additives at higher concentrations [24,25,26]. Numerous synthetic or natural antioxidant agents [27,28,29] and sorbic acid (SA) [30] have been used in formulations of polymeric packaging materials to produce antioxidant and antimicrobial films.

Polypropylene (PP) is a conventional packaging material with extensive utilization due to its ease of processing and good barrier properties [31]. Compared to other synthetic polymers, PP benefits from higher resistance to chemicals, excellent mechanical properties, and stability upon high temperatures during film-making process. Therefore, PP is a suitable base for multifunctional food packaging films. Incorporating BHT and α-tocopherol into PP and low-density polyethylene (LDPE) packaging films has shown that the resulting active films exhibited excellent retention of antioxidants upon storage time. That means that in the PP matrix, α-tocopherol is released slowly, thus, its retention or active time will be longer in the polymeric matrix. Whereas some losses of antioxidants were observed in the case of LDPE matrix [32].

Many studies are reporting on the application of active LDPE/PP films with natural antioxidant such as quercetin and α-tocopherol [33], green tea extract [34], *Allium sativum* essential oil [35]. Furthermore, LDPE active films containing synthetic antioxidants showed stability effect on linoleic acid [32], prevention of lipid oxidation in fish muscle [36], and odour stability in Asadero cheese [37]. Also, the antimicrobial effects of SA, or its salts, have been proved in a polymeric and biopolymeric matrix [38].

However, few researches have addressed the implementation of synthetic polymers such as PP to incorporate active agents for development of multifunctional active packaging. Development of functional packaging films, e.g., antioxidant-antimicrobial films, is one of the novel packaging areas to inhibit microbial growth and to prevent or reduce undesirable deteriorating reactions in order to maintain food quality and extend the shelf-life of packaged foods. We previously designed PP-based antimicrobial films with sorbic acid [8] and antioxidant films containing BHT, BHA and TBHQ [39]. Those works only focused on incorporating single antimicrobial or antioxidant agents at different individual concentrations into the PP-matrix. The main focus of current study is the application of combined antioxidant-antimicrobial substances in the PP films and investigating the physical, mechanical, thermal, structural, antioxidant and antimicrobial properties of the obtained composite active films.

## 2. Materials and Methods

### 2.1. Preparation of Active Films

Polypropylene granules (Marun Petrochemical, Bandar-e-Emam, Iran), BHT and BHA (Merck, Darmstadt, Germany), and sorbic acid (AppliChem, Darmstadt, Germany) were formulated as shown in Table 1. The composite films were formulated based on the optimal amounts of active additives obtained in the previous experiments. First, we produced films containing 2, 4 and 6% antimicrobial agents (sorbic acid) [8], then, 1, 2 and 3% antioxidants (BHA, BHT and TBHQ) films [39]. Different physico-mechanical, thermal, structural, antioxidant/antimicrobial and migration properties of the obtained films were investigated. For this study, the composite films were separately prepared by combination of antioxidant and antimicrobial additives (as shown in Table 1) according to the procedure reported previously [40].

### 2.2. Measurement of Film Properties

#### 2.2.1. Thickness Measurement

A digital micrometre (0–25 mm with an accuracy of 0.001 mm, Mitutoyo, Japan) was used to measure film thickness. Triplicate measurements were performed at different locations of each film specimen (10 × 10 cm^2^) taking a relaxation time of 2 min before the next measurement.

#### 2.2.2. Mechanical Properties

Mechanical properties of the active films were evaluated by measuring the tensile strength (TS, MPa) and elongation at break (E_b_, %) by ASTM D882-18 method [41] using a Tensile Machine (Cometech, Model QC-506B1, Taichung City, Taiwan) with a maximum load cell capacity of 10 kN. Film samples were cut into strips with a 10 cm length and 1 cm width. Tensile tests were carried out at the gauge distance and the rate of grip separation of 50 mm and 10 mm.min^−1^, respectively. Measurements were performed in triplicates. TS (MPa) and E_b_ (%) were calculated using the following Equations (1) and (2),

(1) $$TS=\frac{F_{max}}{A_{min}}$$

(2) $$E_{b}=\frac{L_{max}}{L_{0}}\;\times 100$$

where *F_max_* is the maximum extensional force, *A_min_* is the minimum cross-sectional area, *L_max_* is amount of elongation at the rupture time, and *L*_0_ is the initial length of film sample.

#### 2.2.3. Fourier-Transform Infrared (FTIR) Spectrum Analysis

To determine possible interactions of functional groups between antioxidant additives with PP matrix, FTIR analysis was performed using a spectrophotometer (Bruker, Tensor 27, Bremen, Germany) by scanning wavenumbers in a range of 4000 to 400 cm^−1^ at ambient temperature (25 °C) [42].

#### 2.2.4. Scanning Electron Microscopy (SEM)

To study the cross-sectional microstructure of the composite films a scanning electron microscope (MIRA3, TESCAN, Brno, Czech Republic) was used with a rising voltage of 1 to 15 kV with 20-fold magnification [43,44]. For SEM observations, a dry film samples were frozen in the liquid nitrogen for 10 min and then broken to produce a new cross-section followed by vacuum sputtering with a thin layer of gold. The best images were obtained at a voltage of 5 kV and a 2 µm scale [45].

#### 2.2.5. Dynamic Mechanical Thermal Analysis (DMTA)

Dynamic mechanical and thermal properties of the active composite films were measured using a dynamic mechanical analyser (Triton Technology Ltd., model Tritec 2000 DMA, Keyworth, Nottinghamshire, UK) according to ASTM D5026 [46]. Samples were prepared in an approximate dimension of 9 × 5 × 0.026 mm^3^ (length × width × thickness) and tested at a temperature range of −50 to 150 °C, a sinusoidal frequency of 1 Hz, and 2 °C min^−1^ temperature growths. Changes in storage (elastic or E′) and loss (viscous, E′′) moduli, as well as (loss factor (tan δ, E′′/E′) were measured as a function of time and temperature. These properties provide insights on thermomechanical properties including glass transition temperature (Tg), damping behaviour, and effectiveness of cure [47].

#### 2.2.6. Film Transparency

**Figure 1:**
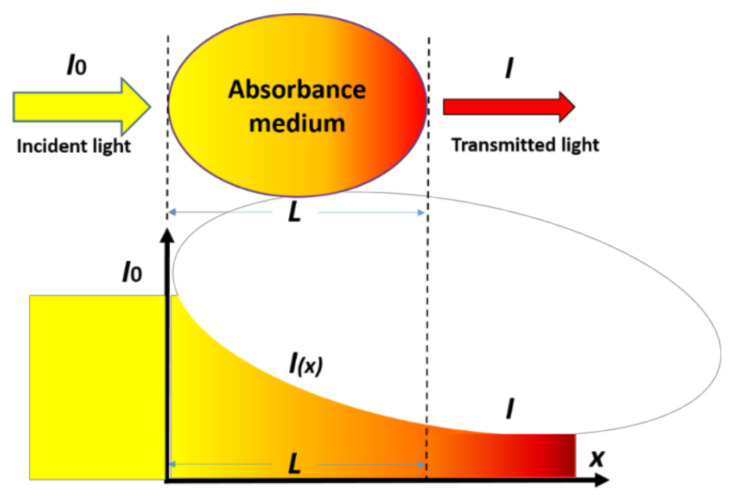
A schematic representation of Beer-Lambert’s law to show relationship between the attenuation of incident light through an object the properties of that object, adopted from Sun and Chuang [48].

The transparency of a film sample relays on its light barrier properties. The relationship between the attenuation of light that falls on an observing medium and the properties of that medium can be explained by Beer-Lambert’s law (Figure 1),

(3) $$A=\log\left(\frac{I_{0}}{I}\right)=\log\left(\frac{1}{T}\right)$$

where *T* is transmittance; *A* is absorbance; *I*_0_ and *I* are the intensity of original light and of the transmitted light, respectively.

In this study, a double-beam spectrophotometer (SPECORD 250 PLUS, Jena, Germany) at a wavelength range between 200 nm and 800 nm was used to measure the UV and visible light barrier properties film samples [49]. The transparency determined by per cent of transmitted light through film specimen was calculated using light transmittance at wavelengths of 600 nm (T_600_) from Equation (4), which was derived from Equation (3),

(4) T = −logT600X

where *T* is the transparency of the samples, *T*_600_ is 600 nm transmittance and X is the film thickness in mm.

#### 2.2.7. Contact Angle

To study the effect of additives used on the surface hydrophilic or hydrophobic characteristics of the polypropylene films, the contact angle of an adhesive droplet (Sessile drop) with film surface was measured. First, films were kept fixed on a plane glass surface by double-sided adhesive paper, and a small amount of distilled water (2 µL) was placed on the surface of films to form a droplet. The contact angles were measured using ImageJ freeware with the plug-in Drop Shape Analysis with calculating the tangent angle at the three-phase (film-drop-air) contact point from the liquid side [50]. All data were performed in triplicate that each of them was the average of two angles of one droplet.

#### 2.2.8. Colour Measurement

The colour properties of film samples, including total colour changes (ΔE), yellowness index (YI), and whiteness index (WI) were measured using a colorimeter simulator. In this device, the sample chamber is designed in a way that the angle of the camera and light source is 45° at distance of 30 cm from the sample. Also, the chamber is insulated against environmental light. Film specimen (10 × 10 cm^2^) were mounted on a white standard paper sheet to take photos using a digital camera with a 10-megapixel resolution (Canon, Japan). Lab mode of Adobe Photoshop software (CC 2014) was used to analyse colour parameters [51] according to the Equations (5)–(7),

(5) $$∆E=\sqrt{{\left(L_{St}-L_{Sm}\right)}^{2}+{\left(a_{St}-a_{Sm}\right)}^{2}+{\left(b_{St}-b_{Sm}\right)}^{2}}$$

(6) $$YI=\frac{142.86\;b}{L}$$

(7) $$WI=100-\sqrt{{\left(100-L\right)}^{2}+a^{2}+b^{2}}$$

where Δ*E* is total colour changes; *L*, a, and b correspond to lightness, greenness/redness, and blueness/yellowness, respectively; *St* and *Sm* represent standard and sample, respectively; *YI* and *WI* are yellowness, and whiteness indices, respectively.

Triplicate measurements were performed in a way that each calculation was an average of five different locations.

#### 2.2.9. Antioxidant Capacity

A search for proper antioxidant agents for food preservation is of interest for their economical usage in food formulations [52,53]. Antioxidant capacity of films can be measured by in situ methods [54]. However, DPPH (1,1-Diphenyl-2-Picrylhydrazyl) radical scavenging activity (RSA) measurement is by far the most commonly used method. To measure the antioxidant capacity, small pieces (0.1 g) of film samples were put in Falcon tubes containing 2 mL of methanol followed by shaking for 60 s. The tubes were kept at ambient temperature for 2 h before analysis. A methanolic solution of DPPH (2 mL 0.1 mM) was then added to the methanol extract (500 μL) followed by a vigorous shaking and keeping for 30 min at dark and ambient temperature. The mixture absorbance (A) was determined using a spectrophotometer (UNICO, UV-2100, Dayton, NJ, USA) at 517 nm. To prepare the control sample the same procedure was performed, except that the film extract was replaced by methanol. DPPH RSA (%) was obtained from the following Equation (8) [33]:

(8) $$\mathrm{RSA}\;\left(\%\right)=\left(1-\frac{A_{\mathrm{sample}}}{A_{\mathrm{control}}}\right)\times 100\;.$$

#### 2.2.10. In Vitro Antimicrobial Properties

*Escherichia coli* (ATCC 25922), *Staphylococcus aureus* (ATCC 29523) and *Aspergillus niger* (ATCC 9029) were selected to assess the in-vitro antimicrobial activity of PP-based composite films according to the procedure explained in details earlier [40].

#### 2.2.11. Migration Behaviour

British Standard EN 1186–1 [55] was used to measure the overall migration of additives in the films into different food simulants. Distilled water, acetic acid in water (3% *v*/*v*) and ethanol in water were (95% *v*/*v*) considered as food simulants for aqueous foods (pH > 4.5), acidic foods (pH < 4.5), and fatty foods, respectively. Details of migration test was previously reported [40].

### 2.3. Statistical Analyses

A factorial design using Minitab 16 software was used to perform the statistical analysis. To determine any significant (*p <* 0.05) difference between the means the one-way ANOVA and Tukey’s multiple range comparison test were carried out.

## 3. Results and Discussion

### 3.1. Film Thickness

The average thickness of the films was 25.63 ± 0.25 μm. This value was considered for the calculations where the film thickness was needed.

### 3.2. Mechanical Properties

**Figure 2:**
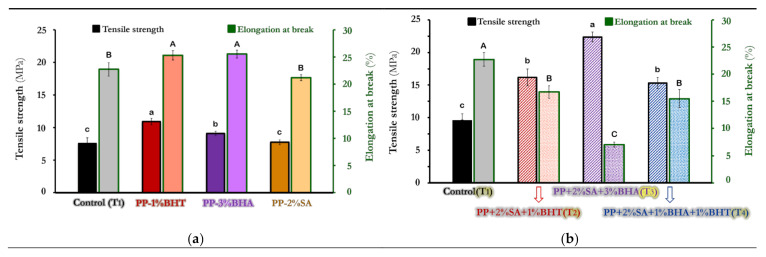
Tensile strength (TS) and elongation at break (E_b_) variations of pure polypropylene (PP) and different active composite films incorporating either single (**a**) or a combination (**b**) of antioxidants/antimicrobial substances. Data are mean of triplicate measurements. Error bars indicate SD values. Different small and capital letters show a significant (*p <* 0.05) difference between means for TS and E_b_ parameters, respectively.

Figure 2 demonstrates the variation of tensile strength (TS) and elongation at break (E_b_) values of control film and composite active films containing either single (a) or a combination (b) of antioxidants/antimicrobial additives.

As shown in Figure 2a, TS and E_b_ values of PP-1%BHT and PP-3%BHA were significantly (*p* < 0.05) higher than those of PP-2%SA and control films, which PP-2%SA film was not significantly different from control. The reason for observed differences among composite films may be attributed to different chemical structure and physical properties of incorporated additives (polarity, molar mass, molar volume and solubility), which could influence the interaction and compatibility of added substances with polymeric matrix [45,56]. This can influence mechanical properties of the composite films. The low polarity of BHT as compared with other additives (polarity: BHT < BHA < SA) may influence BHT compatibility with nonpolar polypropylene [57]. As a result, TS values of composite films followed an order of PP-BHT > PP-BHA > PP-SA (Figure 2a). Besides, the order of molar mass and molar volume was BHT > BHA > SA (Table 2), which may enhance the interaction of larger additives with polymeric matrix. As a result, this can increase TS and E_b_ values of the resulting films. Decreased TS and E_b_ values of SA films compared to those of BHT and BHA films is may be due to the fact that SA is more polar and has lower molar mass compared to BHT and BHA, which this may decrease its compatibility with PP matrix.

As shown in Figure 2b, BHA leads to higher tensile strength in combinatory films. PP-2%SA-3%BHA (T_3_) showed the highest TS (17.91 MPa) and the lowest E_b_ (7.1%) values, followed by T_2_ and T_4_ films, which was not significantly different from each other. It seems that, on one hand, the higher concentrations of BHA, and the possible synergistic effect of SA with BHA, on the other hand, can result in significantly (*p* < 0.05) increased TS and reduced E_b_ values for T_3_ sample. T_2_ and T_4_ films had similarly higher TS, but lower E_b_ values than control. Besides, comparing all combinatory composite films (T_2_–T_4_) (Figure 2b) with those containing individual additives (Figure 2a) reveals that combinatory composite films reveal improved mechanical properties. These results may be explained by possible synergism between dispersed particles in combinatory films. It can be concluded that by combining two or three additives at low concentrations, the same, or even improved, mechanical properties of the film can be obtained.

### 3.3. Fourier-Transform Infrared (FTIR) Spectrum

**Figure 3:**
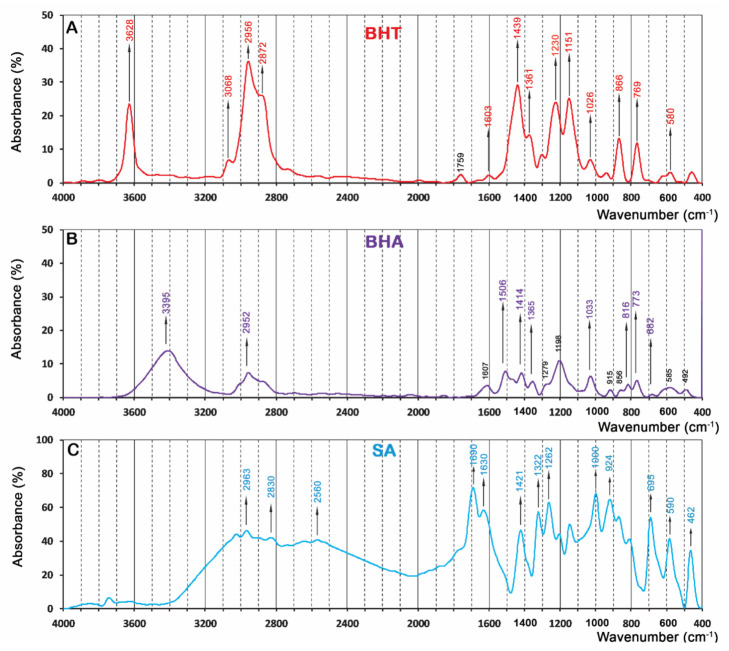
FTIR spectra of butylated hydroxytoluene, BHT (**A**); butylated hydroxyanisole, BHA, (**B**); and sorbic acid, SA (**C**).

FTIR spectra (absorbance % vs. wavenumber or frequency) of BHT, BHA and SA at a wavenumber range of 400–4000 cm^−1^ are presented in Figure 3. BHT’s characteristic absorption peaks (Figure 3A) were at 3628 cm^−1^ (O–H stretching mode of vibration in phenol ring), 3068 cm^−1^ (C‒H stretching), 2956 cm^−1^ (asymmetric vibrations of methyl group), 2872 cm^−1^ (symmetric stretching vibrations of methyl group), 1603 cm^−1^ (C=C bonds stretching), 1439 cm^−1^ (CH_3_ bending vibrations), 1361 cm^−1^ (tert-butyl bending), 1230 cm^−1^ (in plane C‒H bending), 1151 cm^−1^ (in plane O‒H bending), 1026 cm^−1^ (-CH_3_ rocking vibration), 866 and 769 cm^−1^ (C‒C stretching), and 580 cm^−1^ (out of plane phenol ring bending) [56,58,59,60,61]. Also, FTIR analysis revealed the characteristic peaks for BHA (Figure 3B) at wavenumbers (cm^−1^) of 3395 (O–H bound stretching), 2952 (C‒H stretching), 1506 (phenyl ring), 414 (bending of –CH_2_ adjacent to the phenyl), 1365 (bending of –CH_2_ adjacent to the phenyl) and 1033 (C‒O bond stretching), and 815, 765, and 679 (bending of C=C out of plane phenol ring) [62,63,64,65]. Sorbic acid (Figure 3C) showed absorption peaks at wavenumbers (cm^−1^) of 2963 to 2560 (O–H bond stretching modes in a carboxylic acid), 1690 (C=O stretching), 1630 (C=C stretching), 1421 (C–C stretching), 1322 (C–O stretching), 1262 (–CH_3_ rocking vibration), 1000 and 924 (C–C stretching), and 695 to 462 (C=C bending) [40].

**Figure 4:**
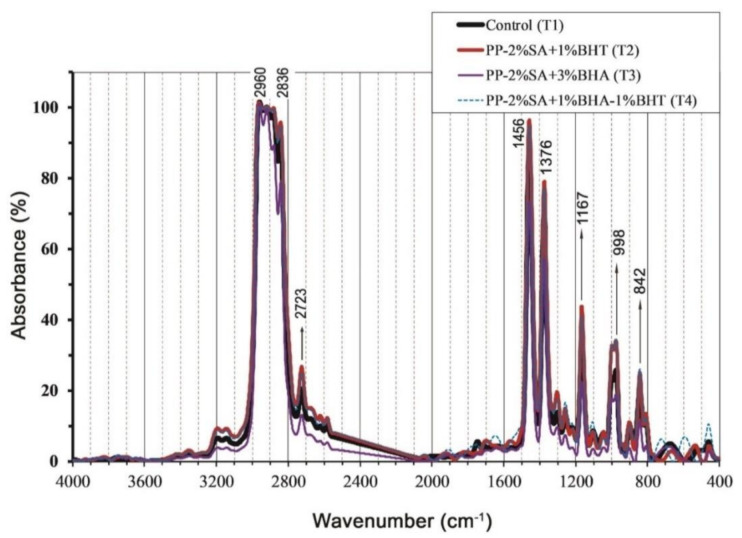
FTIR spectra of pure polypropylene (control, T_1_), PP-2%SA-1%BHT (T_2_), PP-2%SA-3%BHA (T_3_) and PP-2%SA-1%BHT-1%BHA (T_4_) packaging films.

FTIR spectra of pure polypropylene (control, T_1_), PP-2%SA-1%BHT (T_2_), PP-2%SA-3%BHA (T_3_) and PP-2%SA-1%BHT-1%BHA (T_4_) packaging films are shown in Figure 4. Pure PP film as well as active composite films exhibited characteristic absorption peaks at 2960 cm^−1^ (C–H stretching), 2836 cm^−1^ (asymmetric –CH_3_ group’s vibration), 2723 cm^−1^ (C–H out of the plane bending), 1456 cm^−1^ (deformation of the ‒CH_2_ bond), 1376 cm^−1^ (symmetric ‒CH_3_ group’s vibration), 1167 cm^−1^ (C‒C bending related to the backbone of polypropylene), 998 and 842 cm^−1^ (isotactic polypropylene band) [59].

The results of FTIR spectra of composite active films as compared to the control (pure PP) film is shown in Figure 4. As can be seen in this figure, the main components of the FTIR spectrum were detected for all composite films indicating that incorporating BHT, BHA or SA into PP matrix did not lead to significant changes (formation or deletion of new chemical bonds) in the functional groups of PP film. It can be concluded that production of composite films via extrusion process this process leads to physical mixing rather than chemical modification. Similar results were reported by Kang et al. [59] for BHA and BHT, indicating that there is no certain chemical interaction between BHT and PP matrix, thereby, the mixing was mostly physical. From these results, we can speculate that the antioxidants/antimicrobial substances incorporated into the PP matrix have enough mobility to migrate from the film surface into packaged materials.

### 3.4. Scanning Electron Microscopy (SEM)

**Figure 5:**
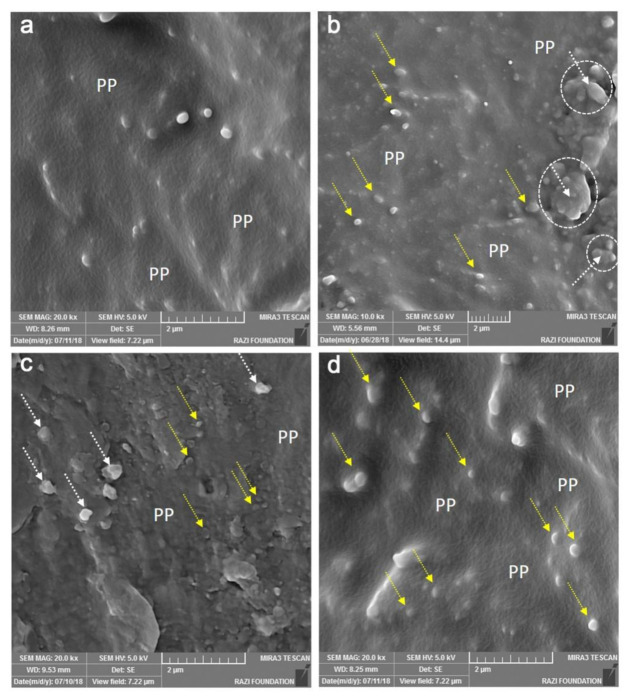
SEM images of pure PP film (control) (**a**) and active composite films: PP-2%SA-1%BHT (**b**); PP-2%SA-3%BHA (**c**); PP-2%SA-1%BHT-1%BHA (**d**).

Scanning electron microscopy is one of the most powerful tools used in various disciplines, with the help of electron bombardment to provide small objects of 10 nm images for study. The SEM images of the produced film samples in a length-scale of 2 μm are shown in Figure 5. The images obtained from the cross-fracture surface of the polypropylene control film (4a) indicated a relatively smooth surface compared to other samples, which is in agreement with Singh et al. [66]. The image 4b represented the surface obtained from the PP-2%SA-1%BHT film (T_2_), which shows both dispersed (yellow arrows) and agglomerated particles (dotted circles and white arrows) of active additives in the polymeric matrix (PP in the figures) showing uneven and coarse cross-section surface of the film. In Figure 5c,d, the background of the polypropylene with visible dispersion of large and small are clearly seen. Among three active film samples, PP-2%SA-1%BHT-1%BHA (T_4_) sample had the most homogenous dispersion of the particles. In the SEM images, it is clear that all particles in the matrix have a size smaller than 0.5 µm, and it seems that the coarse particles (white arrows) are related to SA and the finer particles (yellow arrows) represented antioxidant particles. Since BHA and BHT are melted at temperatures of 48 °C, and 70 °C, respectively (early stages of the film preparation) they are uniformly dispersed in the polymeric matrix (PP) due to the similarity of their structure (lower polarity) with that of the base polymer and appear as fine particles in the SEM images (yellow arrows in Figure 5c). These results are in conflict with those of Goncalves et al. [67], who reported coarse particles of BHT antioxidant in PLA matrix. On the other side, SA has a higher melting point (135 °C) and polarity which results in the lack of compatibility with non-polar PP, hence it may not be melted entirely during film preparation. Similar to Kuplennik et al. [38], where they incorporated potassium sorbate in LLDPE matrix, formed agglomerates led to coarser particles in the matrix.

### 3.5. Dynamic Mechanical Thermal Analysis (DMTA)

**Figure 6:**
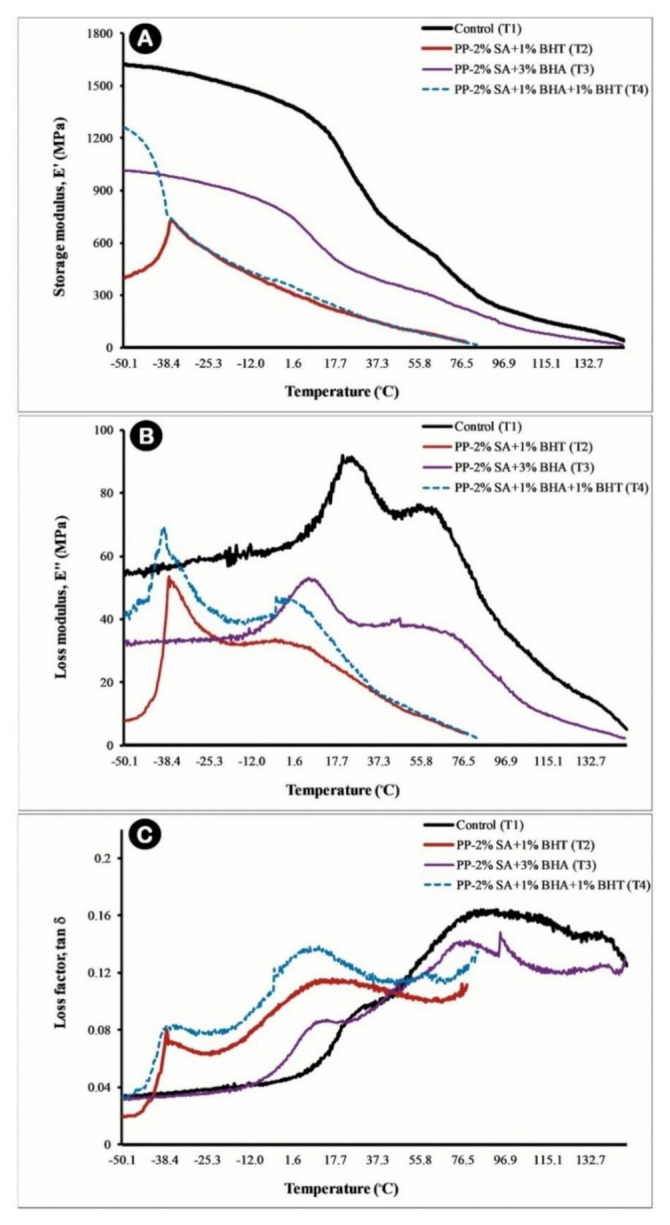
Viscoelastic behaviour for pure polypropylene (control, T_1_) and different active composite films (T_2_–T_4_) as measured by storage modulus, E′ (**A**); loss modulus, E″ (**B**); and loss factor. tan δ, (**C**).

The viscoelastic behaviour of prepared films was tested by DMTA, as well as the measuring glass transition temperature (Tg). The results were shown as a storage modulus (E′), a loss modulus (E″) and a loss factor or damping coefficient (tan δ) as a function of temperature (Figure 6). The E′ relates to the elasticity of the material and is an indicator of its ability to store energy. The E′ changes for pure PP and composite films as a function of temperature (in the range of −50 to 150 °C) are presented in Figure 6A. As can be seen in this figure, the E′ of pure PP film (T_1_) was the highest (1620 MPa) at the test temperature range, and then gradually decreased with increasing the temperature up to 18 °C followed by a sharp decrease. There was an initial increase in the E′ of the PP-2%SA + 1%BHT film at the similar temperature as Tg of the composite. This observation was probably due to the accumulation of large BHT crystals, which, in less than Tg of the composite film, the stress is locked until to reach a sufficient fluidity, and by approaching this temperature, it is provided possibility to move the polymer chains by releasing their trapped energy [38].

The E″ is an indicator of the viscosity of the films and represents the amount of energy lost by the system. As displayed in Figure 6B, in all samples, with increasing temperature, the E″ increased, and after presenting two peaks, dropped again. The sharp increase in the E″ indicated more mobility of the polymer structure because of the glass transition of rubber that was not feasible at temperatures lower than Tg. In the studied temperature range, two transition stages were well observed. The first peak was observed for samples containing BHT at temperatures from −37 to −39 °C, which was related to the β-transition and Tg. In the T_3_ (PP-2%SA-3%BHA) and T_4_ (PP-2%SA-1%BHT-1%BHA) samples (containing 3 and 1% BHA), Tg peaks were depicted at temperatures of 6.7 and −3.9 °C, respectively, which was significantly lower than Tg of pure PP film (25.6 °C). As it could be seen, the shifting of the Tg to lower temperatures could be attributed to the presence of BHT and BHA antioxidants in the polymer matrix, which increased the mobility of polymer chains and accelerated the transition process. The DMTA pattern of PP displayed a second peak at a temperature of 54 °C, which was related to the α transition. This transition was identified as the depreciation of the amorphous polymer chains energy and was found in semi-crystalline polymers [68]. Due to the presence of active materials, the temperature range of the transition was narrowed, which resulted in rising the mobility of polymer chains. Incorporating antioxidant and antimicrobial agents to the PP matrix decreased the peak height of the E″ curve, which was due to the lowering of internal friction, and which indicated a decrease in mechanical energy dissipation [68].

The loss factor (tan δ) is attributed to the equilibrium between the elastic and viscous phase of the material and depends on the mobility of the polymer structure. Based on Figure 6C, tan δ of polypropylene and composites was affected by additives. In a composite containing 3% BHA, the loss factor was less than pure PP polymer at temperatures higher than 47 °C. This could be attributed to limited movement of polymer molecules in the presence of BHA. At temperatures below 47 °C, the addition of BHT increased the loss factor of T_2_ and T_4_ composites. This increase was clearly seen in Figure 6C, which referred to the higher movement range of polymer molecules in the presence of BHT, especially the presence of both antioxidants [69]. According to the results, it was found that incorporating active additives decreased the storage modulus of the films and consequently, their elastic behaviour. This reduction was directly related to the molecular weight of the composite and the particle size. By increasing the amount of low molecular weight additives in the formulation of the film, E′ values decreased. Therefore, the sample containing 2% SA with a molecular weight of 13.1 g·mol^−1^ and 3% BHA with a molecular weight of 160.3 g·mol^−1^ had higher E′ values (at temperature range of −40 to 150 °C). The glass transition was also partly affected by the molecular weight [70]. Therefore, by decreasing molecular weight and by increasing the crystalline portion of composites, Tg, E″, and tan δ were decreased (at temperatures below 47 °C) [68].

In the small amounts of additives in the PP matrix, they acted as a stabilizer and plasticizer, which reduced the Tg and also the melting point that was in agreement with findings of Byun et al. [27]. The results of Ortiz-Vazquez et al. [29] did not show any significant change in Tg by adding 1.5% BHT, and Jamshidian et al. [71] expressed no change in the melting point by adding 1% BHT to the PLA polymer matrix, while Goncalves et al. [67] depicted that the incorporation of antioxidants such as BHT and TBHQ to the PLA increased the free volume of the polymer matrix and consequently the chain mobility, hence Tg, and the melting point decreased, which were in compliance with the results of this study. Overall, it seems that thermal and dynamic and mechanical properties of the active films were most affected by BHT. Therefore, the presence of BHT decreased the storage and loss modulus of the composite films. Moreover, the melting point of the BHT containing film (T_2_) was significantly decreased compared to that of control (T_1_). However, was better in the T_4_ sample made by combining the mixture of three additives, indicating the synergistic effect of the two antioxidants in the presence of antimicrobial agents on the thermal properties of active films.

### 3.6. Film Transparency

**Figure 7:**
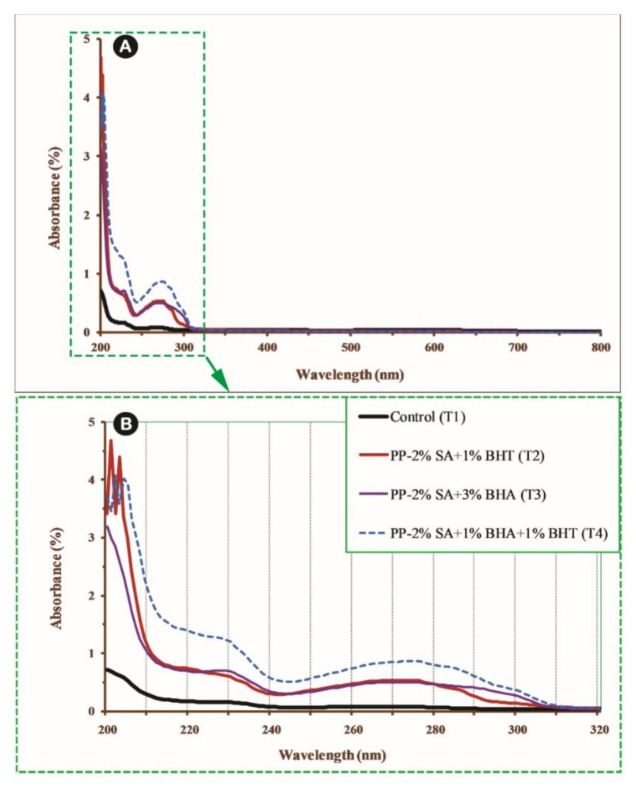
Effect of different composition of additives on UV absorbance of PP-based active films at wavelength range of 200–800 nm (**A**); the same results at wavelength range of 200–320 nm (**B**).

The UV-Vis pattern of the prepared films was illustrated in Figure 7A, (also, in Figure 7B, to show a better clarity at wavelength limit of 200–320 nm).

Control film (T_1_, pure PP) did not show UV absorbance at wavelengths between 200 nm and 800 nm (Figure 7A). However, all active films displayed higher absorbance at wavelength between 200 nm and 300 nm. The samples PP-2%SA-1%BHT (T_2_) and PP-2%SA-3%BHA (T_3_) had almost similar UV absorbance at 200–300 nm (Figure 7B), while the sample PP-2%SA-1%BHT-1%BHA (T_4_) showed higher absorbance that could be explained by Beer-Lambert’s law. The results of the comparison of transparency of the antioxidant and antimicrobial films are shown in Table 3. Incorporating SA and BHT into PP-matrix increased the transparency by about 5%, while it was decreased for other samples; i.e., the transparency in the T_4_ was reduced by 9%. We previously showed that PP-BHT films (at BHT concentrations of 1–3%) had higher transparency values than PP-BHA films (at BHA concentrations of 1–3%). It can be concluded that the higher absorbance of composite films, compared with that of control can be related to higher content of crystalline compounds [72], higher compatibility and uniform distribution of combined additives [45]. This reduced light transmission at a wavelength range of 200–300 nm. Our data are in agreement with earlier reports on optical behaviour of BHT films [73], BHT and BHA in multilayer films (HDPE, EVOH and LDPE) [74] and gelatine-BHT films [45]. The migration of additives from the films is inevitable during storage period, because it should happen to provide antioxidant/antimicrobial effects in the food. There is a possibility that the migration phenomenon can affect the UV absorbance of the film samples, which should be taken into account. 

### 3.7. Contact Angle

Contact angle (θ) is known as an angle between a solid and a liquid or the angle resulting from the fluid on the solid surface. It relates to the wettability of a film surface, which is significant and applicable feature of packaging films considering coatings and printing operations, as well as bacterial adhesion and cleanness of food contact surfaces. The contact angle of the films with a drop of distilled water was considered, and the results are shown in Table 3. The addition of SA, BHA, and BHA did not significantly (*p* > 0.05) change the contact angle, and thereby, the wettability of the composite films. The contact angle obtained for samples varied from 100.15° to 109.03°, which the angle values more than 90° indicated the dominance of the hydrophobic property, and thus, weak wettability of the prepared films [75]. The contact angle of pure PP was 100.15°. Higher contact angle is related to the nature of the hydrophobic polymeric matrix and a slight increased contact angle of the composite films is probably due to the incorporating hydrophobic and water insoluble additives such as SA, BHA and BHT [71].

### 3.8. Colour Attributes

The results of the colour attributes are shown in Table 4. Data indicated that there was no significant (*p > 0.05*) difference in brightness (L*), redness (a*), yellowness (b*), total colour change (∆E), yellowness (YI), and whiteness (WI) indices of composite active films as compared to those of the control. The lower amounts of additives used could account for this result. This was previously confirmed in the case of films prepared by incorporating SA into PP-based antimicrobial films [67].

### 3.9. Antioxidant Properties

Table 5 demonstrates the antioxidant activity of pure PP film and the composite films containing different combinations of BHA, BHT, and SA. As can be seen in this table, DPPH radical scavenging activity of composite films (62–91%) were significantly (*p* < 0.05) higher than that of pure PP film (0.8%). Among different composite film samples, the antioxidant activity of T_4_ sample was the highest (90.8%) followed by T_2_ (81.8%) and T_3_ (62.5%) samples. Antioxidant efficiency and the thermal stability of synthetic antioxidants are different, which may play a role in the observed differences among different composite films. Commercial BHA is a mixture of mostly (90%) 3-BHA (3-tert-butyl-4-hydroxyanisole) and a 10% 2-BHA (2-tert-butyl-4-hydroxyanisole) molecules [76]. The tertiary butyl groups in BHA structure are subjected to different hydroxyl groups making steric barrier, which can lead to reduced antioxidant capacity of BHA, compared with BHT. The presence of such tertiary butyl groups in the BHA chemical structure may alter the antioxidant capacity of phenolic group [71]. It is reported that the butyl steric barrier and the prohibited phenol rings in BHA structure may account for its low thermal stability as well [77]. Overall, this could describe why BHA, containing composite films (T_3_ sample), possesses lower antioxidant capacity, compared with BHT film (T_2_ sample). Surprisingly, as reported in Table 5, composite film with a combination of BHT, BHA and SA exhibited the highest antioxidant activity (a DPPH-RSA value of 90.8%). This can be probably explained by the possible synergism between BHA and BHT molecules, which has also been confirmed in earlier studies [78]. Besides, the presence of sorbic acid in T_4_ film can act as a hydrogen donor resulting the inhibition of free radicals, which has been confirmed by previous studies on the antioxidant action of organic acids [79]. Thus, incorporating SA in combination with BHT and BHA enhanced the antioxidant activity of the resulting composite film (T_4_ sample).

### 3.10. Antimicrobial Properties

The results of microbial experiments of the active films against *E. coli* as gram negative, *S. aureus* as gram positive bacteria, and *A. niger* are shown in Table 5. All active film samples significantly (*p* < 0.05) retarded the growth of *E. coli* and *S. aureus*. Incorporating a 2% concentration of SA in the pure PP matrix reduced the *E. coli* and *S. aureus* counts from 8.70 to 8.56 and from 8.61 to 8.53 (log cfu.mL^−1^), respectively (results are not shown here). As can be seen in Table 5, the addition of the same concentration of sorbic acid in combination with BHA and BHT was more effective than the addition of SA alone. This can be explained by the additional antimicrobial effect of BHT and BHA molecules, as also reported earlier [80]. The antibacterial activity of PP-2%SA-1%BHT-1%BHA (T_4_) sample against both bacteria was more enhanced, compared to PP-2%SA-3%BHA (T_3_) and PP-2%SA-1%BHT (T_2_) active films, due to the synergistic effect of BHT and BHA molecules with each other and with SA. Contrary to what we expected for the anti-mould action of sorbic acid, all active films did not show inhibitory zone in the inoculated *A. niger* cultures. A possible explanation could be the lower thickness of the film samples, in which they fail to release or propagate enough SA molecules into the mould culture to inhibit *A. niger* growth.

### 3.11. Migration Analysis

Table 6 shows the migration behaviour of composite films incorporating antimicrobial/antioxidants additives into different food simulants. The results presented in this table showed no significant differences (*p* > 0.05) in migration behaviour of all composite films in distilled water as simulant for aqueous foods. While, in acidic and fatty food simulants increasing both antioxidants concentration led to a significant (*p* < 0.05) increase in the migration values of the resulting films. Moreover, PP-3%BHA and PP-3%BHT samples exhibited higher migration values than other films in acidic and fatty food simulants. PP-films containing combination of additives at lower concentrations (T_2_ and T_4_ samples), showed lower migration values. The composite films containing BHT showed higher migration values than films containing BHA. This may be explained by difference in their density (ρ), molecular weight (Mv), molecular volume (Mv) and surface area. BHT with higher density (1.048 g·cm^−3^), Mw (220.4 g·mol^−1^), Mv (256 cm^3^·mol^−1^), and surface area (19.4 × 10^−9^ cm^2^·mol^−1^) showed higher migration values compared to BHA with ρ = 1.009 g·cm^−3^, Mw = 180.3 g·mol^−1^, Mv = 182.4 cm^3^·mol^−1^, and surface area = 14.5 × 10^−9^ cm^2^·mol^−1^ [57]. Additives with higher Mw and Mv facilitated the movement of molecules within the polymeric chains, which this would increase their release rate to the simulants [29].

## 4. Conclusions

In this study, PP-based active films containing combined BHA, BHT and sorbic acid additives at different concentrations, were prepared and their physical, mechanical, thermal, structural, antioxidant and antimicrobial properties were investigated. FTIR spectroscopy revealed that the addition of BHT, BHA and SA into PP-matrix was in favour of physical interactions or mixing rather than formation of new chemical bonds or the deletion of existing functional groups. Therefore, the lack of chemical interactions between functional groups might facilitate the release of active antioxidant or antimicrobial agents from film body to the food surface. SEM demonstrated that PP-2%SA-1%BHT-1%BHA (T_4_) sample had the most homogenous dispersion of the particles among three active films, indicating that a combinatory use of these additives at low concentrations produced smooth film surface as the control film. In line with these observations, colour measurements also showed that incorporating antioxidant and antimicrobial additives did not significantly change the colour attributes of the active composite films compared to those of control. However, dynamic mechanical thermal analysis (DMTA) was mostly influenced by the presence of BHT in the composite films, leading reduced storage and loss moduli of the films; therefore, weak viscoelastic behaviour of the films, and lowered melting point. Moreover, the active films demonstrated higher tensile strength (TS) and lower elongation at break (E_b_) values compared to pure PP-film. It was observed that BHT and BHA in the presence of SA, increased and decreased the transparency of the active composite films, respectively. BHT films showed more UV absorbance than BHA films. The addition of SA, BHA and BHT did not significantly affect the contact angle, and thus, the wettability properties of the films. All active films had higher antioxidant and antimicrobial activities than those of pure PP-film. Active films significantly retarded gram-negative and gram-positive bacteria growth. Different properties of the prepared composite active films revealed their potential use for food packaging applications. In relation to the importance of expanding food shelf-life, and the negative impact of the direct application of preservatives on health, active composite PP films used in this study could be useful for food packaging applications suggesting T_2_ and T_4_ samples for cold packing (packaging of food products with low filling temperatures) due their lower thermal properties and T_3_ film for hot packing (with high filling temperatures).

## Figures and Tables

**Table 1:** The sample codes and formulation of active antioxidant/antimicrobial films.

Film Samples	PP	SA (phr)	BHT (phr)	BHA (phr)
Pure PP (control) (T_1_)	100	-	-	-
PP-2%SA-1%BHT (T_2_)	97	2	1	-
PP-2%SA-3%BHA (T_3_)	95	2	-	3
PP-2%SA-1%BHT-1%BHA (T_4_)	96	2	1	1

PP: Polypropylene; SA: sorbic acid; phr: part per hundred rubber; BHT: butylated hydroxy toluene; BHA: butylated hydroxy anisole.

**Table 2:** Chemical structure and physical properties of SA, BHA and BHT.

	SA	BHA	BHT
Chemical structure	**C_6_H_8_O_2_**	**C_11_H_16_O_2_**	**C_15_H_24_O**
	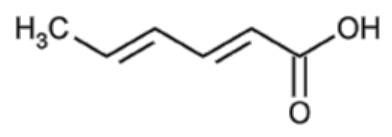	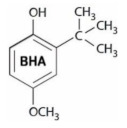	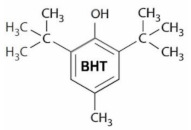
Density (g·cm^−3^)	1.204	1.06	1.048
Solubility in water (g·L^−1^)	1.6	0	1.1
Solubility in ethanol (g·L^−1^)	12.9	Freely soluble	Freely soluble
Molar mass (g·mol^−1^)	112.1	180.2	220.4
Molar volume (cm^3^·mol^−1^)	109.4	182.4	256.0
Polarity	High	Medium	Low

**Table 3:** The results of contact angle and the transparency of polypropylene (PP)-based active films.

Film Samples	Transparency (%)	Contact Angle (θ^°^)
Pure PP (control) (T_1_)	76.80 ± 0.001 * ^b^	100.15 ± 2.20 ^a^
PP-2%SA-1%BHT (T_2_)	80.57 ± 0.004 ^a^	108.15 ± 6.30 ^a^
PP-2%SA-3%BHA (T_3_)	75.23 ± 0.007 ^c^	107.24 ± 1.67 ^a^
PP-2%SA-1%BHT-1%BHA (T_4_)	69.85 ± 0.003 ^d^	109.03 ± 3.60 ^a^

* Data are mean of triplicate measurements ± SD. Different letters in superscripts in each column show significant (*p <* 0.05) differences between means.

**Table 4:** Colour attributes of different polypropylene (PP)-based active films.

	L *	A *	B *	∆E	YI	WI
Pure PP (control) (T_1_)	63.7 ± 0.61 * ^a^	−8.1 ± 0.99 ^a^	−4.8 ± 1.25 ^a^	6.4 ± 1.04 ^a^	−3.2 ± 0.86 ^a^	60.5 ± 0.58 ^a^
PP-2%SA-1%BHT (T_2_)	62.3 ± 1.46 ^a^	−8.8 ± 1.31 ^a^	−5.7 ± 2.72 ^a^	6.7 ± 2.64 ^a^	−3.9 ± 1.88 ^a^	60.7 ± 1.45 ^a^
PP-2%SA-3%BHA (T_3_)	63.2 ± 0.60 ^a^	−9.7 ± 0.31 ^a^	−4.7 ± 0.23 ^a^	6.6 ± 0.16 ^a^	−3.2 ± 0.18 ^a^	61.6 ± 0.53 ^a^
PP-2%SA-1%BHT-1%BHA (T_4_)	62.9 ± 0.50 ^a^	−9.7 ± 1.75 ^a^	−2.2 ± 1.59 ^a^	6.6 ± 1.72 ^a^	−1.5 ± 1.11 ^a^	61.5 ± 0.36 ^a^

* Data are mean of triplicate ± SD (each replicate was an average of 5 measurements from different points on film surface. Different letters in superscripts in each column show significant (*p* < 0.05) differences between means.

**Table 5:** The results of antioxidant capacity expressed as 2,2-diphenyl-1-picrylhydrazyl (DPPH) radical scavenging activity (RSA), and antimicrobial properties of PP-based composite films incorporating a different combination of antioxidants/antimicrobials.

Film Samples	DPPH-RSA (%)	Microbial Analysis		
		*E. coli* (Log cfu/mL)	*S. aureus* (Log cfu/mL)	*A. niger* (Inhibition Area, cm^2^)
Pure PP (control) (T_1_)	0.83 ± 0.12 *^,^^d^	8.70 ± 0.02 ^a^	8.61 ± 0.00 ^a^	0
PP-2%SA-1%BHT (T_2_)	81.84 ± 1.02 ^b^	8.41 ± 0.01 ^b^	8.40 ± 0.01 ^b^	0
PP-2%SA-3%BHA (T_3_)	62.47 ± 1.53 ^c^	8.38 ± 0.01 ^bc^	8.38 ± 0.00 ^b^	0
PP-2%SA-1%BHT-1%BHA (T_4_)	90.81 ± 2.04 ^a^	8.35 ± 0.01 ^c^	8.20 ± 0.01 ^c^	0

* Data are mean (*n* = 3) ± standard deviations. Means with different letters in each column are significantly different (*p* < 0.05).

**Table 6:** Migration behaviour of composite films incorporating single or a combination of antimicrobial/antioxidant additives in aqueous, acidic and fatty food simulants.

Film Samples	Aqueous Foods (pH > 4.5)	Acidic Foods (pH ≤ 4.5)	Fatty Foods
Pure PP (control) (T_1_)	0.78 ± 0.09 * ^b^	0.74 ± 0.06 ^d^	0.78 ± 0.09 ^g^
PP-2% SA	1.95 ± 0.69 ^a^	4.62 ± 0.33 ^a^	2.09 ± 0.39 ^f^
PP-1% BHT	1.45 ± 0.90 ^a^	1.99 ± 0.21 ^c^	4.21 ± 0.17 ^e^
PP-2% BHT	1.76 ± 0.59 ^a^	3.38 ± 0.45 ^b^	5.46 ± 0.27 ^d^
PP-3% BHT	1.96 ± 0.25 ^a^	4.96 ± 0.33 ^a^	7.96 ± 0.35 ^a^
PP-1% BHA	1.38 ± 0.12 ^a^	2.09 ± 0.21 ^c^	4.62 ± 0.33 ^e^
PP-2% BHA	1.96 ± 0.66 ^a^	3.98 ± 0.16 ^b^	5.84 ± 0.27 ^d^
PP-3% BHA	1.99 ± 0.63 ^a^	5.22 ± 0.26 ^a^	8.54 ± 0.26 ^a^
PP-2%SA-1%BHT (T_2_)	2.32 ± 0.39 ^a^	2.12 ± 0.23 ^c^	6.37 ± 0.30 ^c^
PP-2%SA-3%BHA (T_3_)	2.84 ± 0.44 ^a^	3.66 ± 0.36 ^b^	7.22 ± 0.17 ^b^
PP-2%SA-1%BHT-1%BHA (T_4_)	2.43 ± 0.50 ^a^	2.36 ± 0.28 ^c^	6.64 ± 0.16 ^c^

* Data expressed as mg·dm^−2^ are average of triplicate measurements ± standard deviations. Mean values with different letters in each column are significantly different (*p* < 0.05).

## Data Availability

The data presented in this study are available in the article.
